# Enhancing equitable engagement for digital health promotion: Lessons from evaluating a childrearing app in Indonesia

**DOI:** 10.1177/20552076231222112

**Published:** 2023-12-25

**Authors:** Victoria Loblay, Mahalakshmi Ekambareshwar, Aila Naderbagi, Yun JC Song, Michele Ford, Iqthyer Zahed, Adam Yoon, Ian B Hickie, Haley M LaMonica

**Affiliations:** 1 Brain and Mind Centre, 4334 The University of Sydney, Gadigal country, Sydney, NSW Australia; 2 Faculty of Arts and Social Sciences, 4334 The University of Sydney, Gadigal country, Sydney, NSW Australia

**Keywords:** Complexity science, community psychology, socioemotional development, culture, parenting, digital health, mHealth, user engagement

## Abstract

Part of the appeal of digital health interventions, including mHealth, is the potential for greater reach in places where conventional health promotion is hampered by geographical, financial or social barriers. Yet, ‘engagement’ – typically understood as user experience and interactions with technology - remains a persistent challenge, particularly in places where technology access or familiarity with technology is limited. We undertook an evaluation of a childrearing app to promote socioemotional and cognitive development in early childhood across the world. In this article, we present findings from qualitative research on app rollout in Indonesia, the first of numerous low- and middle-income countries targeted by the app. We draw on systems theory and complexity thinking to broaden the lens of ‘engagement’ beyond individual users to encompass collective systems (families and communities), exploring how the intervention was harnessed to meet local contextual needs. The qualitative research involved semi-structured interviews, workshops and audio diaries with 57 diverse stakeholders, including Indonesian parents, caregivers, and collaborators involved in funding, development, and dissemination of the app. We observed the importance of social connection, sense-making, and interactive learning for enhancing engagement with the app and its messages. Enthusiastic users, strongly linked across community networks (e.g. kindergarten teachers), improvised dissemination strategies to facilitate uptake. Interactive learning that tapped into familiar social structures (e.g. intergenerational hierarchies) was crucial for engagement. Understanding ways the app failed to tap into structures of social connection served to highlight the need to embed strategies to support collective engagement.

## Background

Digital health promotion is increasingly seen as a promising avenue for addressing health challenges facing children and young people, including healthy development in the early years. With the widespread adoption of smartphones, mobile health (mHealth) interventions, such as caregiving/parenting apps to promote optimal cognitive and socioemotional development, represent possible alternatives to complex multicomponent and face-to-face parenting programs.^1^ A particular advantage of utilising digital tools is their potential for greater reach in contexts where health promotion and provision of health services are hampered by geographical, financial, or social barriers, including in low- and middle-income countries.^2,3^

However, realising this vision for digital health promotion is often thwarted by low uptake and reluctance among target populations to use the tools.^2,4^ Challenges and limitations associated with low uptake are well documented and include factors such as time constraints, accessibility of devices and Internet, privacy concerns, financial burdens (e.g. cost of data), sociocultural, language or literacy issues.^5,6^ Less well understood is how to address these challenges, particularly for mHealth technologies implemented in community contexts in culturally diverse, low-resource settings.^7^

Participatory co-design and user-testing methods involving a range of stakeholders have been identified as important to improve usability and engagement with end products through iteratively developing and adapting technologies.^8^ Yet, issues involving implementation and sustainment are not usually considered in design processes.^9^ Moreover, despite exponential growth in mobile app-based health promotion, only a small fraction of health apps are subject to rigorous evaluation.^3^ Scale-up and spread of mHealth interventions also lead to unpredictable processes and outcomes through interaction with local contextual circumstances.^10^ Longer-term evaluation approaches are therefore critical for understanding change processes associated with scale-up and spread of digital health interventions and informing ongoing sustainability.

### Evaluation approaches to inform scale-up and spread of mHealth interventions

Conventional thinking and evaluation of digital health interventions have tended to produce evidence focusing on the assessment of individual-level outcomes such as behaviour change or knowledge gain. Focusing solely on whether intended audiences replace old behaviours with new ones, or use knowledge they have gained, risks overlooking key factors that influence engagement including power, language, and dynamics of knowledge movement in particular contexts.^11^ The thorny issue of ‘engagement’ in mHealth is frequently understood in terms of individuals’ interaction with technology^12^ and gamification^3^ such that measuring engagement becomes a matter of mining user analytics. Yet engagement with digital interventions stretches well beyond technology use, embedding within collective settings (e.g. families, workplaces or schools) and becoming part of conversations, routines or generating new roles within communities^13,14^ Diverse forms of engagement may be particularly important to understand in low-resource contexts where devices may be shared and technology access or familiarity with technology may be limited. In order to enable equitable engagement, we need evaluation approaches capable of capturing the breadth and diversity of change processes, as well as unpredictable outcomes.

Complexity science offers one such alternative for evaluating mHealth interventions. Though digital health interventions often appear as a single component (e.g. mobile-based app) even seemingly simple interventions may have multiple interactions with their contexts.^15^ The greater the complexity of the contextual setting into which the technological intervention is introduced, the less likely it is to be adopted, scaled up, spread or sustained.^16^ In this sense, complexity is better understood as a property of a system, rather than an intervention.^17^

Where interventions are introduced within complex systems (e.g. schools, social media communities or early childhood education networks), the system will adapt to changes brought about by the intervention.^17^ Intervention components interact with one another, such as when marketing strategies influence how app users view the purpose of an mHealth intervention. Contextual features of the system, such as widespread availability of internet access or lack thereof, also reshape interventions. In this sense, digital health interventions are not fixed in their form. When put into practice, digital tools shift shape as they are ‘made to matter’, continually producing multiple effects.^18^ This means change will be non-linear, making it difficult to discern for evaluators in the early phases of an intervention. Evaluative evidence, then, is ‘emergent, contingent and multiple’ and interventions are capable of remaking context*.*^18^

Understanding the role of context, and the dynamics between context and intervention, is critical for complexity-informed approaches to evaluation. With interventions such as childrearing apps that seek to use and change relationships, it is important to understand existing networks and relationship dynamics within households and communities, as well as to track how the intervention transforms and interacts across social structures and networks in local contexts.^19^ Complex adaptive systems thinking brings the focus to the actions of agents and patterns in the system. Instead of assessing intervention effectiveness through measuring the acquisition of skills or knowledge, the evaluation attends to the capability of local change agents to adapt the intervention to satisfy unmet needs arising from unique local circumstances.^20^

The task for evaluators is therefore to understand and constructively deal with variations that arise across diverse contexts. One way to accomplish this is to identify and leverage instances of ‘self-organisation’^10^ in which local actors harness digital tools to facilitate spread and engagement with the intervention as needed in local contexts. In this article, we draw on qualitative data from an evaluation of a childrearing app in Indonesia. We explore self-organisation in practice by examining how local actors made sense of and creatively adapted the intervention to meet local contextual needs. We describe emergent change processes that arose through applying an evaluative lens across two contextual systems: families/households and community networks. In each system, we consider (a) how the app intersected with relationship dynamics (i.e. between mothers and fathers, between caregivers and children, across generations and communities) and (b) new conceptions of cultural value that accompanied encounters with the app. By tracking ways unfamiliar knowledge, ideas and practices were made familiar, and the particular circumstances where the app gained momentum – or not – we seek to better comprehend the contextual dynamics that support self-organisation and engagement with this digital health intervention.

## Context: the Thrive by Five International Program in Indonesia

The Thrive by Five International Program is an initiative of Minderoo Foundation, an Australian philanthropic organisation, that aims to promote the importance and understanding of childhood development in the early years. The Program aspires to develop and disseminate content for parents and caregivers of children aged 0–5 years across the world, with a specific emphasis on low- and middle-income countries.^21^ At the time of publication, Thrive by Five has been implemented in Indonesia, Malaysia, Afghanistan, Uzbekistan, Kyrgyzstan, Namibia, Kenya, Cameroon, and the Democratic Republic of the Congo. Drawing on anthropological and neuroscientific research, as well as co-design processes with local experts, parents and caregivers, Thrive by Five content is adapted according to language, culture and values of each country.^8^ The content comprises ‘Collective Actions’ that contain suggestions for childrearing activities and scientific content explaining why activities are important for children's development.^22^ Content is primarily delivered through the Thrive by Five app, available free through app stores in each country and tailored to other digital (e.g. WhatsApp) and non-digital dissemination methods (e.g. pamphlets, cards and workshops) in particular contexts.

Based on strategic partnerships within the Minderoo Foundation network, Indonesia was selected as a proof-of-concept country for the initial launch of the Thrive by Five International Program.^8^ Central to the development of Thrive by Five – known as *Cilukbalita* (peekaboo) in Indonesia – has been the collaborative engagement with Indonesian parents and caregivers, local partner organisations and experts in psychology, early childhood education and curriculum development, social work, medicine, nutrition and international policy initiatives to support the development of content that accounts for cultural nuances of the Indonesian context, as well as ethnicity, geography, socioeconomic status and religion. Two local partner organisations supported the co-design, development, launch and dissemination of Thrive by Five in Indonesia: AIMI (Asosiasi Ibu Menyusui Indonesia), a non-profit organisation that aims to increase knowledge and practice of breastfeeding in Indonesia,^23^ and The Indonesian Child Welfare Foundation (YKAI), an organisation committed to improving living conditions for underprivileged children through health and education projects.^24^ As the lead organisation for the Thrive by Five International Program, Minderoo Foundation has played an instrumental role in the development and dissemination of the app and its content, working closely with software development partners, as well as coordinating partnerships to develop marketing, communications and dissemination strategies that underpinned the rollout of Thrive by Five in Indonesia.

Implementing an mHealth intervention such as a childrearing app in Indonesia typifies many of the challenges outlined above. Characterised by vast geographic and cultural diversity, there are over 1300 discrete cultural communities and enormous variation in attitudes to childrearing across the archipelago. The value placed on children is reflected in the love and attention given to them from families, neighbours and community members, especially in early childhood. Children are generally raised by mothers, grandmothers, older sisters and other female relatives, as well as female domestic workers.^25^ However, studies point to the seriousness with which men consider their engagement in their children's lives.^26^ Much of the research examining variations in values and attitudes to childrearing has focused on the Javanese, Indonesia's majority ethnic group. Research across different social classes has found social pressures to demonstrate respect towards superiors are more typical among upper-class Javanese.^27^ While older children are encouraged to conform to characteristic ideals of collectivism such as social harmony and displaying obedience and respect toward elders, research suggests young Javanese children (under age 5 or 6) are generally seen as unable to understand social expectations and treated with permissiveness and indulgence.^28^ They are also described as having few opportunities to develop initiative and dependence on parents may be encouraged.^29^ Less emphasis is placed on respectful obedience among rural farming families, with greater tolerance for self-reliance and responsibility among children.^27^

In terms of contemporary childrearing in Indonesia, some regions remain relatively isolated, while others are increasingly connected to globalised cosmopolitan discourses on childrearing and early childhood development. Indonesia has a burgeoning digital landscape with 78% of Indonesians using mobile phones for Internet access^30^ and digital technologies leveraged to improve health care services. Apps and websites provide general health information such as pregnancy guidance^31^ and advice on parenting challenges.^32^ Despite popularity of web-based sources for health information, there is a marked urban–rural divide in digital connectivity.^33^ A key challenge for implementing Thrive by Five in Indonesia has therefore been to reach and engage rural communities, as well as areas with low literacy and education rates.

## Methods

### Study design

A comprehensive overview of the broader evaluation study for the Thrive by Five International Program is described elsewhere.^34^ The evaluation study is a multi-country, mixed-methods evaluation of the program. The mixed-methods approach in Indonesia included quantitative surveys with 158 caregivers at 2 timepoints (16 and 24 weeks after the launch of the app) between July and September 2022. At each timepoint, a series of semi-structured interviews (*n*  =  14) and workshops (*n* = 6) were conducted via Zoom with a total of *n* = 47 parents and caregivers. From 18 to 22 weeks post-launch, two citizen ethnographers recorded audio diaries. We also spoke with *n* = 10 collaborators including personnel from the Minderoo Foundation, AIMI and YKAI. The findings reported here are from qualitative research (interviews, workshops, audio diaries) that aimed to gain an in-depth understanding of how parents and caregivers, including early childhood educators, were incorporating the Thrive by Five app and content into their everyday lives. We also sought to understand implementation processes associated with the Thrive by Five rollout in the Indonesian context, including the experiences of local collaborators and key stakeholders involved in funding, development and dissemination of the app.

YKAI organisation recruited local caregiver participants for the qualitative evaluation through YKAI branches across 15 provinces (Bali, West Nusa Tenggara, South Sumatra, Aceh, Riau Islands, East Java, West Java, Central Java, Yogyakarta, Jakarta, Banten, East Kalimantan, Central Kalimantan, South Sulawesi, West Papua). Site coordinators at each branch recruited participants through their own community network of Thrive by Five app users, using word-of-mouth until the target sample size was achieved. Further demographic information on interview/workshop participants is provided in Table 1. All participants provided prior written and verbal consent and were reimbursed for their time. Qualitative and demographic data was deidentified and stored separately from consent records. To preserve independence of the evaluation and maintain confidentiality, YKAI was not involved in data collection or analysis.

**Table 1.** Workshop and interview participant demographics.

	16 weeks post-launch (July 2022)	25 weeks post-launch (Sept 2022)
	*n* (%)	*n* (%)
*47 caregivers*		
Region		
Lesser Sunda Islands		
Bali	0 (0)	5 (11)
West Nusa Tenggara	1 (2)	3 (6)
Sumatra		
South Sumatra	7 (15)	0 (0)
Aceh	1 (2)	0 (0)
Riau Islands	0 (0)	1 (2)
Java		
East Java	0 (0)	1 (2)
West Java	0 (0)	1 (2)
Central Java	8 (17)	0 (0)
Yogyakarta	0 (0)	1 (2)
Jakarta	0 (0)	6 (13)
Banten	0 (0)	1 (2)
Kalimantan		
East Kalimantan	8 (17)	0 (0)
Central Kalimantan	0 (0)	1 (2)
Sulawesi		
South Sulawesi	1 (2)	0 (0)
West Papua	1 (2)	0 (0)
Caregiver role		
Mother	23 (49)	16 (34)
Father	0 (0)	0 (0)
Grandparent	2 (4)	2 (4)
Sibling	0 (0)	1 (2)
Uncle/aunt	2 (4)	0 (0)
Early childhood educator*	2 (4)	6 (13)
Education level		
Primary/high school	10 (21)	5 (11)
Diploma	3 (6)	0 (0)
University degree	14 (30)	15 (32)
*82 children between 47 caregivers*		
Age of children		
0–1 years	6 (7)	5 (6)
2–3 years	7 (8)	4 (5)
3–5 years	14 (17)	4 (5)
5 years +	20 (24)	22 (27)

* Early childhood educators occupied multiple caregiver roles (e.g. parent and educator).

### Qualitative data collection

Qualitative interviews (60–90 min) and workshops (2 h) were conducted via Zoom and recorded in full, connecting researchers in their Sydney homes with participants in the midst of their day-to-day activities across different regions and contexts in Indonesia. Participants’ cameras provided glimpses of preschool classrooms, home office spaces, coastal palm trees and village housing. Interviews and workshops were led by two female postdoctoral researchers (VL, ME), both with extensive experience conducting qualitative interviews with parents in relation to health practices. Two additional postdoctoral researchers (AN, IZ) were also present as note takers during the interviews. Cultural and linguistic differences between the research team and participants were mediated by a translator fluent in English and Bahasa Indonesia and certified by NAATI (Australian translation certifying authority), who translated questions posed in English. Efforts were made to use the same translator throughout, achieved for all but one interview and one workshop. After each interview/workshop, the research team debriefed with the translator to clarify points of confusion and engage in ‘sense-making of the collective experiences’.^35^ English questions and translations were transcribed verbatim by AN and IZ and augmented with reflexive fieldnotes from all researchers present at each research event.

Through interviews and workshops, we identified highly engaged participants and invited them to train as citizen ethnographers to record audio diaries, which have been shown to be an effective way to add greater depth to data.^36^ A Zoom training session run by VL, an ethnographer experienced in participatory ethnographic methods, supported participants to ask questions and understand topics of interest for the evaluation. Cues and ethnography tips were provided to encourage them to reflect on and document their day-to-day experiences in detail. Following the training, two citizen ethnographers from rural areas recorded 3 hours audio diaries over 4 weeks. Audio diaries were translated as audio recordings by the translator and transcribed in full. They produced a glimpse of everyday challenges and triumphs of family life and the range of ways participants were applying Thrive by Five*.* In this sense, audio diaries provided an unstructured format for gathering evaluative evidence to capture the elusive and personal aspects of what happens during interventions.^37^

### Statistical and qualitative data analysis

In this article, our interest is in how the Thrive by Five childrearing app was harnessed to meet particular local needs within the Indonesian context. We explore how Thrive by Five intersects with family and community dynamics, as well as cultural values and attitudes to childrearing. Descriptive statistics have been used to characterise the sample based on demographic data in Table 1. All other data analysis was qualitative in nature. Following Tsing,^38^ we treat our evaluation research encounters as ‘dialogues across difference’, in which we collaboratively explore the purpose, meaning and value of the app with our research participants to engage in interactive evidence-making. This sensitised our analytical lens to attune to ‘creative qualities of interconnection across difference’.^38^ Our analysis does not proceed from the assumption that the app is a ready-made intervention whose design and cultural adaptation is built-in. Rather, we focus on the range of ways app users shape and remake the mHealth intervention, generating multiple forms of meaning, dissemination and engagement with the app.

Analytic synthesis of qualitative data was an iterative process involving multiple stages. Initial coding of interview/workshop data was a sense-making exercise to tease out thematic patterns relevant for the evaluation, conducted by VL and ME using NVivo software (Version 14).^39^ The analysis also drew on the country expertise of MF for cultural interpretations and contextual background. Audio diaries were analysed by two researchers (VL, AN) with methodological expertise in ethnography. Each independently wrote reflective fieldnotes and audio-diary stories that expanded on themes from interviews/workshops were written up as case studies. The final stage of analysis involved VL drawing on principles of thematic analysis^40^ across the entire qualitative data set, focusing on social processes and emergent meanings arising from Thrive by Five interactions.

## Results

Our findings capture a variety of geographical, social and cultural experiences of childrearing from different regions across Indonesia. Our analysis explores these diverse interactions with Thrive by Five in terms of the social processes and emergent meanings that take shape in relation to three social systems: (a) household and family dynamics, (b) systems of cultural value and (c) community networks. To protect the identity of participants, the findings are presented according to six broad regional areas designated as follows: Lesser Sunda Islands (Bali, West Nusa Tenggara); Sumatra (South Sumatra, Aceh, Riau Islands); Java (East Java, West Java, Central Java, Yogyakarta, Jakarta, Banten); Kalimantan (East Kalimantan, Cental Kalimantan); Sulawesi (South Sulawesi); and West Papua.

### Situating Thrive by Five within household and family dynamics

Understanding how Thrive by Five app use is situated within structures of connection in households and across broader family relationships is crucial for appreciating the uptake of the app in family contexts. Important aspects of this include gendered caring roles and normative divisions of labour relating to childrearing. Intergenerational dynamics around the ‘right’ way to bring up children, particularly where grandparents are intensively involved in caregiving, also play a significant role in shaping the use of Thrive by Five.

#### Navigating Thrive by Five in relation to gender roles and household divisions of labour

Though neither fathers nor grandfathers volunteered to participate in the study, our in-depth conversations with participants overwhelmingly conveyed that mothers and grandmothers generally bear the main responsibility for childrearing in Indonesia. Nevertheless, some mothers told us how their husbands were very involved in using the app:

As a couple, it's not only my responsibility to take care of the child, it's also his responsibility. If he has free time, he asks the kids, ‘Let's all try this, let's do the activity.’ So, he's had a positive impact … My husband is quite helpful. If I sum it up, the only thing he couldn’t do for children is breastfeeding. He does the rest of it. Also, he is very diligent in taking care of the kids. Now, using the app, we are having very structured, manageable children.

(Mother, Java)

This university-educated participant had left work to stay home with her children, and her husband was also fully engaged in parenting responsibilities. The app served to structure their shared approach to childrearing. Other mothers also described sharing household responsibilities and childcare with their husbands. In each of these cases, it appeared content sharing was *preceded* by a mutual sense of responsibility, rather than initiating a shift in gendered roles.

One mother, a health professional shouldering most of the childrearing responsibilities, was surprised when asked whether she involved her husband in app activities:

In the instructions it says to do activities with ‘your family’… when you [interviewer] ask me, ‘Does the father join in the activities in the app?’ I thought, ‘Oh yes he probably should join!’ It didn’t cross my mind.

It was not until the researcher questioned the father's involvement that this mother realised the app content could apply to her husband. She told us, ‘for Indonesian mothers like me’, it would be helpful if the content encouraged them to do activities ‘with the father and mother’. It had not occurred to her that doing things with the ‘family’ also meant with the father:

Probably because I feel like I take care of everything, so it didn’t cross my mind to ask him to join me.

(Mother, Java)

Many female caregivers considered app messages to be something they were responsible for applying within their families, particularly in terms of additional time spent playing and connecting with children. In a rural village, a school-educated mother who provided support to parents in the community told us she was making an effort to spend more time with her children since using the app. But this was accompanied by an emotional realisation:

After I downloaded and used the app, if I may say, I felt quite devastated. I realised that I am working eight hours a day outside with communities, doing all of these activities with mothers from other households and I was neglecting my own children.

(Mother, Kalimantan)

In this way, new knowledge could introduce new burdens in terms of juggling multiple responsibilities. Notably, this mother had help from her younger sister who cared for her children whilst she worked, but had not considered sharing the app or activities with her.

#### Thrive by Five as a tool for negotiating intergenerational caregiving

In our conversations with caregivers, we heard how grandparents were often intimately involved in the raising their grandchildren. Many grandparents cared for children while parents worked and therefore had more time to do app activities. One grandmother explained how she passed on learnings to her adult daughter, who was too busy to use the app:

After I use the app, I discuss it with my daughter or with the nanny… so I say [to them], ‘Do you know that you have to do this?’

(Grandmother, Java)

We also heard of intergenerational differences between the way parents and grandparents managed challenging behaviour. In a workshop with working mothers, many in the group described deliberately using the app to encourage grandparents to shift approaches to children's behaviour. They told us the ‘scientific information’ in the app meant grandparents would ‘believe it more’:

With this app we can show them [grandparents] that there is a reason why screentime is not good. If it is just us [parents] telling them that screentime is not good without having something to show them, they would just ignore what we say.

(Mother, Kalimantan)

The convenience of having a digital tool from a trusted source enabled these mothers to present information with scientific authority that reinforced messages they were already trying to convey to grandparents.

In a rural area workshop, participants recounted how grandparents would care for their children during the busy tobacco season. The grandparents did not own mobile phones, so parents would tell grandparents about app content they had been learning. We heard how the grandmothers had become ‘even more careful in taking care of them’ than the parents:

After using the app, we [parents] have learnt that… we should make sure the environment where [the children] play is safe, say from sharp objects for example, and we conveyed this information to the grandparents. [Now]… if there is an instance in which we let the children play without checking for sharp objects or things that are dangerous, the grandparents would reprimand us, saying, ‘Why didn’t you check? This is what you told us we should be doing!’ So we get told off sometimes.

(Early childhood educator & mother, Lesser Sunda Islands)

In these ways, app and content usage intersects with intergenerational transitions and power dynamics around childrearing. Where parents are unable to care for their children while working, they are still able to direct *how* their children are cared for. Further, app messages may be used by both parents and grandparents to assert authority and remind others of the ‘right’ way to approach childrearing.

### Emerging values and attitudes to childrearing

A primary objective of Thrive by Five is to support the cultural adaptation of the app and align content with cultural values and traditions relating to childrearing.^8^ The evaluation therefore aimed to ascertain caregivers’ perspectives on the cultural relevance of the app and content, and the extent to which they felt it fostered connections to cultural traditions and values. Dialogues with participants on the topic of culture elicited a range of responses. Often these were straightforward affirmations of the app's cultural appropriateness. One caregiver, who was studying early childhood education, picked up on the philosophy of responsive, nurturing care that implicitly guides the content, explaining how it fit well with their cultural approach:

Our culture teaches that you have to do good things for your children. One of those things is being gentle with them.

(Early childhood educator and mother, Sumatra)

Another participant, a school-educated community worker from a rural area, took a different view:

If you think about our culture … usually the way we would get our children to behave is by scaring them about things. And, in the app, it talks about how we need to say good things, and we tell them things gently. So, I would say it's very opposite of what we usually do culturally.

(Mother, Kalimantan)

Caregivers did not always perceive traditional values and approaches to childrearing as something they wanted to instil in their interactions with their children. One mother, a university graduate caring for her children full-time, explained she did not want to encourage dependence in her children, viewing it as ‘spoiling’:

When they are a kid or baby, they don’t do anything. You don’t let them do anything. If they need something, they would say, ‘Mum, can you help me with this? Mum, can you help me get that?’

(Mother, Java)

Rather than repeat the mistakes of her parents’ generation, this mother wanted her children to ‘be more independent’ and be someone ‘who helps others’. She proudly told us how, through applying app content, her child would now get her things or do things for her when asked. Other caregivers similarly described how since using the app, their children were more responsive, obedient and respectful. Meanwhile, concepts such as independence coalesced with familiar cultural ideals of helping others and respect for elders. This demonstrates how cultural ideals continually shift and adapt as app messages mingle with multiple sources of knowledge about the best way to raise children.

**Figure 1.**
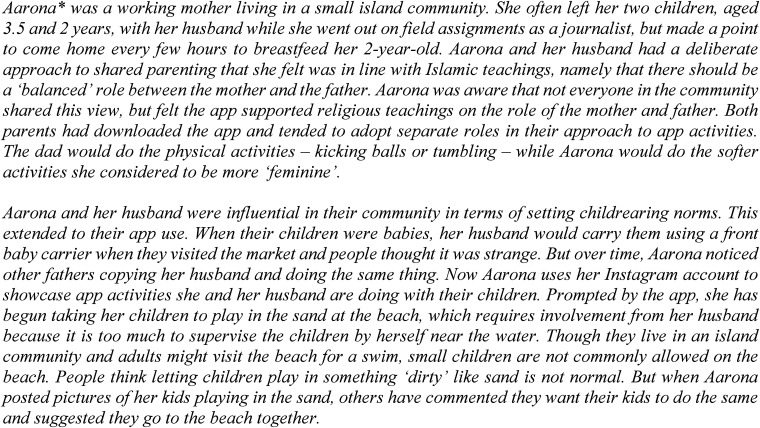
Case study, Sumatra region.

In some instances, caregivers drew on their interpretations of Islamic doctrine as they negotiated concepts and new knowledge from the app. In the following case study (Figure 1), we outline the story of a couple from the Sumatra region who were shaping cultural norms through a blend of traditional religious teachings, app-inspired activities and social media.

### Evolving networks of interaction in local communities

A striking theme in discussions with expert collaborators was that Indonesians liked to learn interactively and that engagement with the app hinged on its ability to facilitate ways for people to learn together:

The app tells you to learn things by yourself. In Indonesia, we have a strong interest in early childhood education, but we do it together so it is more enjoyable. It is different with this app, you have to do it yourself.

(Expert collaborator)

This sentiment was also reflected in discussions with caregivers and repeated requests for the app to incorporate a question/answer feature. And yet, even in the absence of explicit features enabling interactivity, we encountered a number of enthusiastically engaged users who initiated creative approaches to content dissemination that encouraged interaction. This was usually accomplished through harnessing a variety of community networks. As seen above in the case study, a walk through a village marketplace with a father holding his baby may trigger other men in the community to adopt new caregiving practices. And social media feeds could facilitate conversations around new activities and approaches to play.

In another rural village in Lesser Sunda Islands, a grandmother had taken it upon herself to spread app messages within the local kindergarten where she worked. This grandmother recruited other kindergarten staff to download and use the app, not only with children in the classroom but also within families. She encouraged kindergarten parents to download the app and told us the mothers were showing real interest. This grandmother had a vision that these mothers could share the app with their husbands, mostly farmers, who could teach their kids whilst working in the rice paddy fields, enabling ‘directed parenting, not just do-whatever-you-want kind of parenting’. However, she also repeatedly stressed the need for the app to incorporate the ability to interact with other users, which would be more interesting ‘because we can see culture from other regions’.

In rural Kalimantan region, we heard from a mother who was a community worker, working with other parents. She confessed she had not used the app extensively, but it was evident that she was widely communicating a handful of messages. She had been telling parents she worked with to start reading to their children, something that was not widely practised across Indonesia. She had also used handwashing advice from the app to bring a diarrhoea outbreak in a local kindergarten under control.

## Discussion

The rollout of the Thrive by Five International program in Indonesia, as the first proof-of-concept country, provides rich lessons that are both particular to the local region and instructive for conceptualising effective implementation of digital health promotion interventions more generally. With support from YKAI, we engaged in interactive evidence-making mediated by technologies such as Zoom, smartphone voice-recorders and survey software. The participants were mostly proficient in Bahasa Indonesia, with the exception of two workshops where some participants were not able to fully articulate their thoughts and discussions were mediated by other participants. Despite the limitations of relying on technological access and translation, however, we were able to have extensive conversations with a wide range of participants that conveyed diverse experiences of technology and childrearing. Notably, there were difficulties recruiting male participants and the vast majority of participants were female caregivers, reflecting the emphasis on female caregiving in Indonesia noted earlier. Nevertheless, in-depth lines of questioning provided insight into the roles of male caregivers and how the app and activities were shared with other family members.

The reluctance of fathers to participate in this evaluation is mirrored in other studies endeavouring to recruit parents or ‘mothers and fathers.’^41^ It also reflects the broader challenge of engaging users who may benefit most from Thrive by Five. Our evaluation addresses this challenge by illustrating patterns of change and knowledge movement that foster engagement across families and diverse communities, including rural areas with low digital connectivity. We observed the importance of social connection and interactive learning for enhancing enthusiasm for the app and its messages. Where structures of social connection were missing, such as where users were unable to learn interactively through a question/answer forum or ‘seeing’ what other users were doing, there was a sense of missed opportunity. Conversely, enthusiastic users who were strongly linked across community networks were able to act as intermediary change agents,^42^ as seen when a community worker used new ideas about handwashing to intervene in a diarrhoea outbreak at a local kindergarten or where a kindergarten teacher brainstormed ways to encourage local farming families to change their approach to parenting. These change agents harnessed their networks to integrate digital intervention into the social fabric of the community and magnify the impact of app messages.

Similarly, sharing the app across family networks demonstrates how it can be used to mitigate potential areas of intergenerational conflict about the ‘correct’ way to bring up children. The digital intervention could be used to diffuse arguments about screen time. And grandparents, even in the absence of direct access to smartphones, could be instructed about ways to keep the children safe. It is similarly telling that grandparents were still able to reassert their dominance in the family hierarchy by reprimanding their adult children for not adequately complying with content about children's safety. This demonstrates how new knowledge gained through the digital intervention is melded and shaped through pre-existing social dynamics and intergenerational power relations. It also fits with broader trends identified in the use of digital media to obtain health-related information and the active role that users play as ‘cocreators’ of digital knowledge^43^ through curating, sharing and sometimes reformulating digital content from the ground up.^44^

Identifying and attending to these patterns of self-organisation and interdependencies through which people interact and make sense of an intervention are vital for improving scale-up and spread.^10^ Conversation and interactive learning offer opportunities for sense-making that have been recognised as an important enabler of the success of interventions.^45^ Sense-making allows local actors to improvise and adapt interventions so that information and new knowledge are not simply passed on without change, but integrated into relationships and clarified against old beliefs.

The evaluation interviews sometimes provided a forum for sense-making in relation to the app. When we asked a mother about whether she had shared the app with her husband, the question prompted her to reflect differently on app instructions to do activities with the ‘family’. Roles around caregiving were so entrenched, that it had not occurred to her that ‘family’ included her husband. Her impressions indicate that explicit instructions to involve ‘fathers’ may be a necessary trigger to encourage their engagement with app activities. The interview exchange further illustrates the crucial role of sense-making and dialogue across differences for shifting awareness about how family and gender roles could expand beyond the current status quo in ways that could be supportive for both children and adults.

Similarly, evaluation questions about how the app and its content supported local cultural practices sometimes encouraged participants to consider how app activities related to traditional values and approaches to childrearing. Their responses revealed the versatility and multiplicity of concepts of culture and how cultural ideals around notions such as independence are continually evolving through encounters with new knowledge and experiences. Though Indonesia is frequently characterised as collectivist, this did not preclude participants from wanting to cultivate independence in their children, a finding that has similarly been observed in past research with Javanese families.^27^ In our evaluation, the value of independence was articulated not only in terms of doing things for oneself but also in terms of being someone who helps others, demonstrating how conceptions of independence are not mutually exclusive with collectivist ideals. The blending of seemingly incompatible ideas to forge new forms of compatible knowledge has been described by Tsing^38^ as characteristic of cultural production that happens with the ‘friction’ of global interconnections across difference. In this sense, concepts of culture are not a priori assumptions but emerge through interactions between app users, technologies, social discourse and evaluation conversations.

It is important, however, to recognise that not all forms of self-organisation and sense-making necessarily lead to constructive change. One working mother had absorbed a new sense of individual responsibility for implementing app content within her family. Even though other family members were also caring for her children, reading app messages about the importance of bonding and playing with children had prompted this mother to perceive she was ‘neglecting’ her children whilst she helped other families in the community, something she felt ‘devastated’ about. Deborah Lupton^46^ has critiqued the implicit rationale of digital health interventions whereby users are assumed to be individual ‘atomised’ actors, who are able to responsibly fulfil the designated requirements of ‘self-care’. This focus on the individual, she argues, both reduces health challenges to individual problems while simultaneously neglecting the centrality of relationships and communal groups in affecting people's health.^46^ In the context of childrearing, research has emphasised that parents are more likely to partake in effective caregiving roles in communities that see raising children as a collective responsibility.^47^ Though the response of this working mother was not widely repeated in our evaluation research, it signals the potential risk of burdening users through conveying additional caring responsibilities and implicitly encouraging one-to-one caregiving. One way to address this risk may be to craft content that reinforces the value of interdependencies around collective caregiving and encourage sharing the app or its messages across different family members.

From an evidence-making intervention perspective,^18^ we see how evidence and intervention impacts are continually made, not only as users engage with app technology, but as they integrate and adapt new ideas and learnings with their families, communities and even through evaluation interview dialogues. Anthropological research on mHealth and smartphones in diverse cultural contexts suggests that the adaptation and development of these technologies have moved from professional designers to public users.^44^ This has implications for how we conceive of the concept of cultural adaptation in relation to the tailoring of digital health interventions, whereby adaptation is perhaps better understood as an unpredictable and creative process of collaborative production of cultural models of value that continues to emerge as interventions evolve. Recognising processes of cultural adaptation of interventions that take place as people interact with technologies also highlights the limits of neat metaphors of ‘spread’ and ‘adoption’ of interventions.

## Conclusion

Realising the promise of equitable reach of mHealth interventions such as childrearing apps demands an appreciation of nuanced change processes that extend beyond knowledge gain, behaviour change or individual modes of engagement. Focusing only on how individuals interact and engage with technology in mHealth interventions obscures collective modes of engagement that happen through sense-making and social interaction. Drawing on complexity thinking can help broaden our analytical lens to encompass a systems perspective, in which micro-changes are considered in tandem with interactions across contextual systems – in this case families and communities – leading to emergent adaptations. Longer time frames for evaluation and methods to promote sense-making conversations will allow a better appreciation of the multiplicity and diversity of change processes that take place within collective systems. In order to encourage sustained momentum and equitable engagement, local change agents need to be identified and empowered through opportunities for interactive learning. Over time, ‘simple rules’ about what drives self-organising could be identified, providing guidance for optimising digital health interventions in complex systems.^48^ Some of these processes are already underway in our project. After presenting evaluation findings to local partner organisations in Indonesia, community leaders immediately began to consider new ways to strategically include fathers in rural community forums for learning about childrearing. Such initiatives are just one of many potential new directions for Thrive by Five in Indonesia and signal the generative potential of evaluative evidence.

## Supplemental Material

sj-docx-1-dhj-10.1177_20552076231222112 - Supplemental material for Enhancing equitable engagement for digital health promotion: Lessons from evaluating a childrearing app in Indonesia Click here for additional data file. Supplemental material, sj-docx-1-dhj-10.1177_20552076231222112 for Enhancing equitable engagement for digital health promotion: Lessons from evaluating a childrearing app in Indonesia by Victoria Loblay, Mahalakshmi Ekambareshwar, Aila Naderbagi and Yun JC Song, Michele Ford, Iqthyer Zahed, Adam Yoon, Ian B Hickie, Haley M LaMonica in DIGITAL HEALTH

sj-docx-2-dhj-10.1177_20552076231222112 - Supplemental material for Enhancing equitable engagement for digital health promotion: Lessons from evaluating a childrearing app in Indonesia Click here for additional data file. Supplemental material, sj-docx-2-dhj-10.1177_20552076231222112 for Enhancing equitable engagement for digital health promotion: Lessons from evaluating a childrearing app in Indonesia by Victoria Loblay, Mahalakshmi Ekambareshwar, Aila Naderbagi and Yun JC Song, Michele Ford, Iqthyer Zahed, Adam Yoon, Ian B Hickie, Haley M LaMonica in DIGITAL HEALTH

sj-docx-3-dhj-10.1177_20552076231222112 - Supplemental material for Enhancing equitable engagement for digital health promotion: Lessons from evaluating a childrearing app in Indonesia Click here for additional data file. Supplemental material, sj-docx-3-dhj-10.1177_20552076231222112 for Enhancing equitable engagement for digital health promotion: Lessons from evaluating a childrearing app in Indonesia by Victoria Loblay, Mahalakshmi Ekambareshwar, Aila Naderbagi and Yun JC Song, Michele Ford, Iqthyer Zahed, Adam Yoon, Ian B Hickie, Haley M LaMonica in DIGITAL HEALTH

sj-docx-4-dhj-10.1177_20552076231222112 - Supplemental material for Enhancing equitable engagement for digital health promotion: Lessons from evaluating a childrearing app in Indonesia Click here for additional data file. Supplemental material, sj-docx-4-dhj-10.1177_20552076231222112 for Enhancing equitable engagement for digital health promotion: Lessons from evaluating a childrearing app in Indonesia by Victoria Loblay, Mahalakshmi Ekambareshwar, Aila Naderbagi and Yun JC Song, Michele Ford, Iqthyer Zahed, Adam Yoon, Ian B Hickie, Haley M LaMonica in DIGITAL HEALTH
