# Supplementary material for: Enhancing equitable engagement for digital health promotion: Lessons from evaluating a childrearing app in Indonesia
Source: Digit Health. 2023 Dec 25;9:20552076231222112. doi: 10.1177/20552076231222112 (PMC10752113; doi:10.1177/20552076231222112)
Supplement: sj-docx-3-dhj-10.1177_20552076231222112 - Supplemental material for Enhancing equitable engagement for digital health promotion: Lessons from evaluating a childrearing app in Indonesia [file sj-docx-3-dhj-10.1177_20552076231222112.docx]

**Semi-structured interview schedule - collaborators (site specific research team, local subject matter experts, other key stakeholders)**

| 1 | Role and responsibilities | - Can you tell us a bit about your organisation and   your usual role?   - How long have you been working in the organisation? - What experience do you have in working with parents and early childhood development? |
| --- | --- | --- |
| 2 | Partnership story | - In what ways have you been involved with the Thrive by Five app? - How did your organisation become involved with the Thrive by Five app? - Can you tell us about your experiences with the implementation of the app within your country?   *Prompt to describe planning process if it doesn’t come up*   - What role has your organisation played in the implementation of the app? - Were there other people or organisations that assisted with the implementation of the Thrive by Five app? Can you describe their role/involvement? - Has your involvement with Thrive by Five led to new partnerships or relationships with other organisations or colleagues in your country?   *Prompt to describe* |
| 3 | Value add and fit with existing activity | - What does Thrive by Five add to what you are already doing in this area? Why is that important? - Did you learn anything new from your engagements with the Thrive by Five app? Has this changed how you approach childhood development concepts? - What other resources do you provide parents to support early childhood development? - What resources are available to people with low literacy, or those who don’t have access to the internet or mobile phones? How does Thrive by Five compare to these? |
| 4 | Dissemination experiences | - Do you use Thrive by Five as part of your professional practice? How so? - Do you suggest parents/caregivers try the app?   *If yes:* Can you describe how parents respond to the Thrive by Five app?   - Were some parents/caregivers hesitant to sign up to the app? Why? - Has anyone told you about their experience of using the app? *Prompt to describe* |
| 5 | Implementation context | - Based on your experience with implementation of the Thrive by Five app, what were some of the key factors that influenced the implementation of the app within your country? - Were there training processes that supported the implementation of the app? If so, can you describe your experience of training? - Were there any other projects/activities that took place at the same time as the implementation of the Thrive by Five app? Did they facilitate and/or inhibit implementation of the app? |
| 4 | Evaluation | - Did your organisation set goals in relation to implementation of the Thrive by Five app?   *Prompt to describe*   - Were you involved in the testing and design phases of the app? Describe your experience of this process and what was your contribution? - Was there anything you think should have been done differently in terms of app implementation and support to parents? |
