# Supplementary material for: Enhancing equitable engagement for digital health promotion: Lessons from evaluating a childrearing app in Indonesia
Source: Digit Health. 2023 Dec 25;9:20552076231222112. doi: 10.1177/20552076231222112 (PMC10752113; doi:10.1177/20552076231222112)
Supplement: sj-docx-4-dhj-10.1177_20552076231222112 - Supplemental material for Enhancing equitable engagement for digital health promotion: Lessons from evaluating a childrearing app in Indonesia [file sj-docx-4-dhj-10.1177_20552076231222112.docx]

**Evaluation Workshop Agenda**

**Introduction**

- Introductions – Name and roles
- Overview of workshop
  - Experience: Quality, acceptability and usability of Thrive by Five
  - Impact: How has Thrive by Five affected your approach to parenting

**Demographics** (collected via the chat function in Zoom)

1. How old are you?

Text: _________________

1. Are you a mother or father of a child 0-5 years?
   - Mother
   - Father
   - Other

*If Q2 is ‘Other’ go to Q3*

*If Q2 is ‘Mother’ or ‘Father’ go to Q4*

1. Are you a caregiver, close relative, or close friend of a child between 0-5 years?
   - Caregiver (e.g., [insert common term for domestic caregiver])
   - Close relative (e.g., aunt, grandparent)
   - Close friend
   - Other
2. How many children do you care for under the age of 5?

Text: __________________

1. How many years of full-time education have you completed?

Text: __________________

**Thrive by Five**

- How frequently do you use the app?
- Which of the activities have you enjoyed the most or found to be most effective?
- Which of the activities have not been beneficial?
- Have you shared the information you’ve learned from Thrive by Five with anyone (e.g. spouse, friends, teacher)? If so, how did they feel about the ideas you shared?
- Do you use Thrive by Five with other family or friends? In what ways? Did you find any activities challenging to introduce in your family?
- Was the content of Thrive by Five relevant to you? Did you find the content culturally appropriate? Did any of the activities challenge values that are important in your culture?

**Confidence and connection**

- Has the information provided by Thrive by Five changed your sense of confidence as a caregiver?
- Has Thrive by Five influenced how you think about what is best for your child?
- How did the scientific content make you feel about trying new activities and making decisions?
- How has Thrive by Five affected your relationship with the child/ren you care for?
- Did the Thrive by Five activities affect the child/ren’s connections to others in the family?
- Did the Thrive by Five activities affect the child/ren’s connections with the broader community?

**Knowledge gain**

- What did you learn from Thrive by Five?
- Was the scientific content in Thrive by Five easy to understand? How did it affect your understanding of childhood development?
- Was the content consistent with what you have learned about parenting and child development from other caregivers, doctors or health professionals, or the media?
- How has that new knowledge impacted on your parenting?
- In what ways have you changed the way you interact with the child/ren you care for?
- What new knowledge has had the biggest impact on your relationship with the child?
- Is there additional information you wish had been in the app? Are there gaps in the content or information that you think would help you be the best parent you can be to your child?

**Anthropological and cultural impact**

- What cultural factors are reinforced through Thrive by Five?
- Does Thrive by Five allow cultural representation of self/self-actualisation empowerment?
- Does Thrive by Five support a broadening of the definition of family? Did the content support a collectivist approach to parenting for you and the child/ren you are for?
- Do the knowledge and activities in Thrive by Five result in a shift in community thinking?
- Does Thrive by Five help you feel more empowered to do what you know is good for children?
- Do you feel Thrive by Five is for the communal good?
- Does Thrive by Five enable the sharing of cultural knowledge on land as a community and does it reinforce connection to language, land, and country?
